# Supplementary figures and images for: The inflated mitochondrial genomes of siphonous green algae reflect processes driving expansion of noncoding DNA and proliferation of introns
Source: PeerJ. 2020 Jan 3;8:e8273. doi: 10.7717/peerj.8273 (PMC6944098; doi:10.7717/peerj.8273)

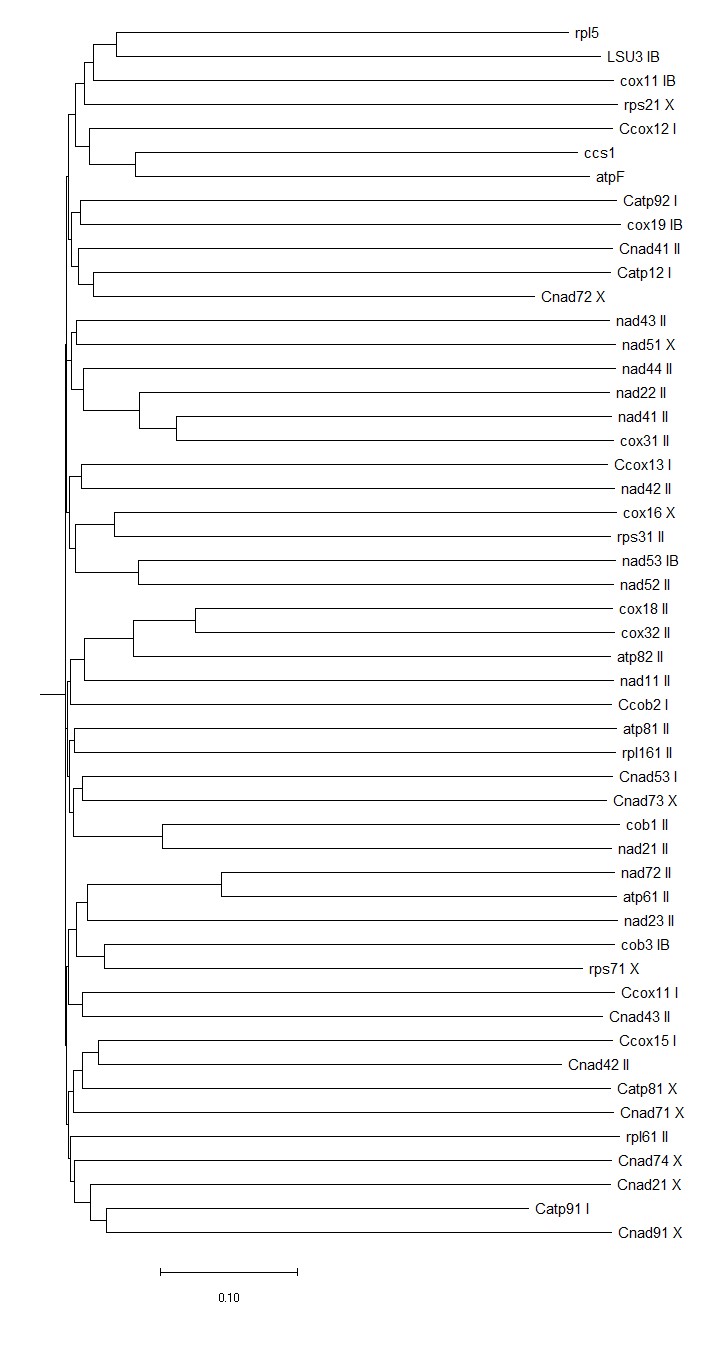

Supplement: Supplemental Information 1 [file peerj-08-8273-s001.jpg]
