# Supplementary material for: The inflated mitochondrial genomes of siphonous green algae reflect processes driving expansion of noncoding DNA and proliferation of introns
Source: PeerJ. 2020 Jan 3;8:e8273. doi: 10.7717/peerj.8273 (PMC6944098; doi:10.7717/peerj.8273)
Supplement: Supplemental Information 2 [file peerj-08-8273-s002.pdf]

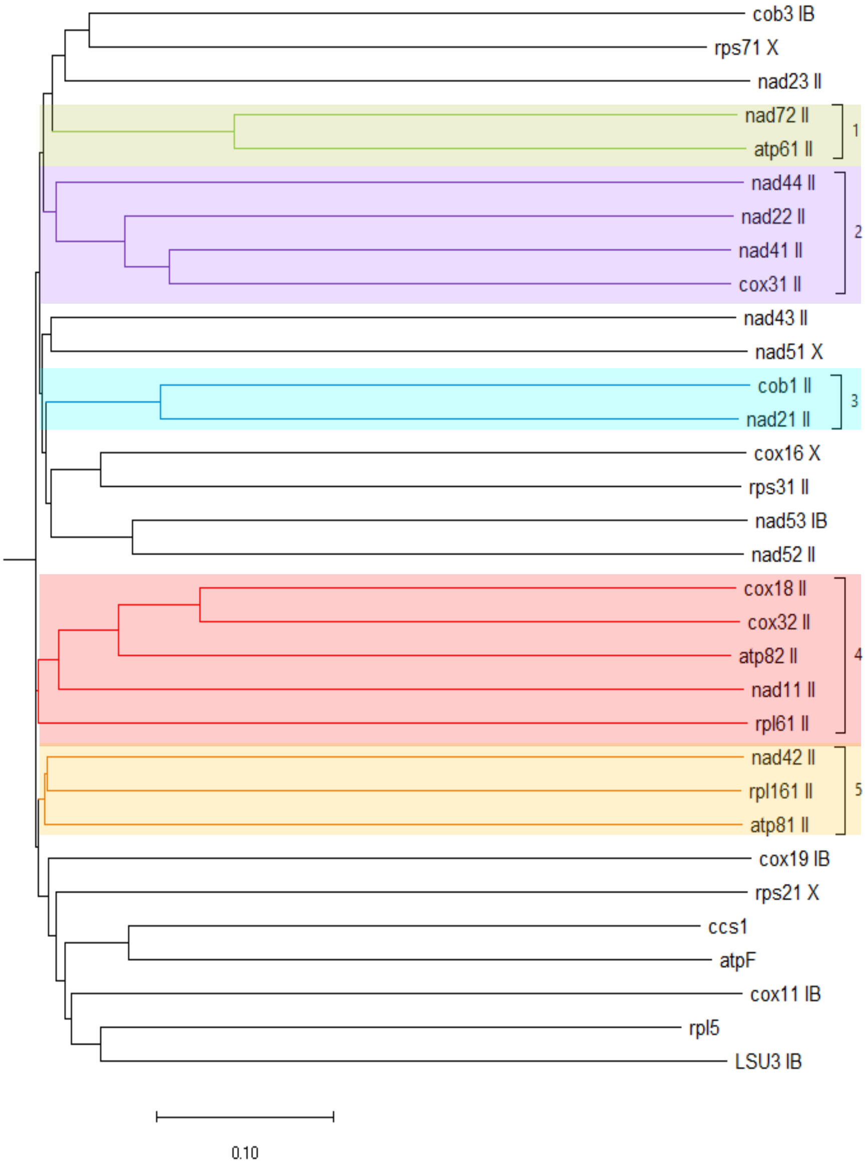

Neighbour joining tree from Clustal Omega distance matrix of ORF-lacking introns in *Ostreobium quekettii* mitochondrial and chloroplast genomes, and MAFFT alignments corresponding to clusters.

Group 1:

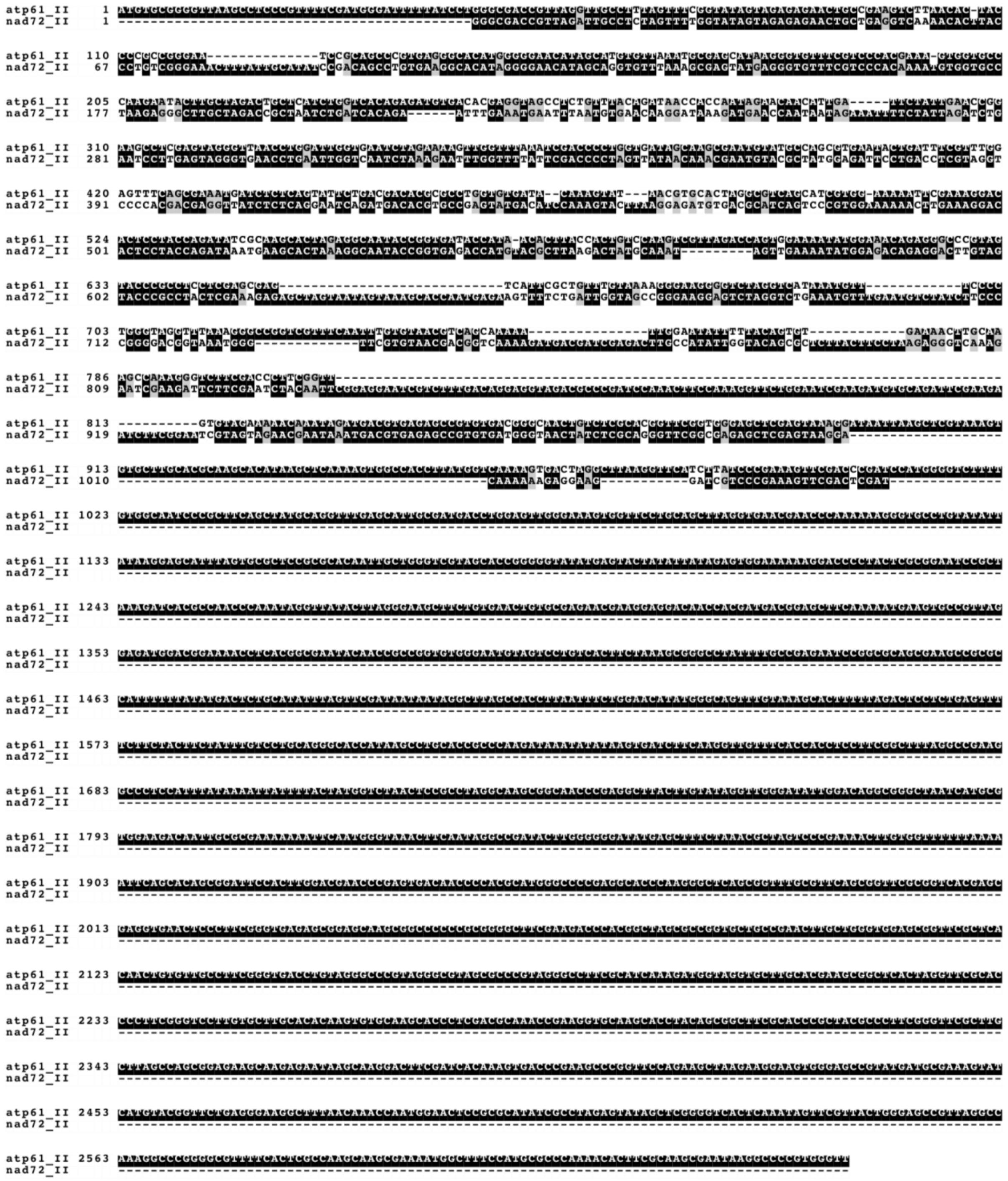

Group 2:

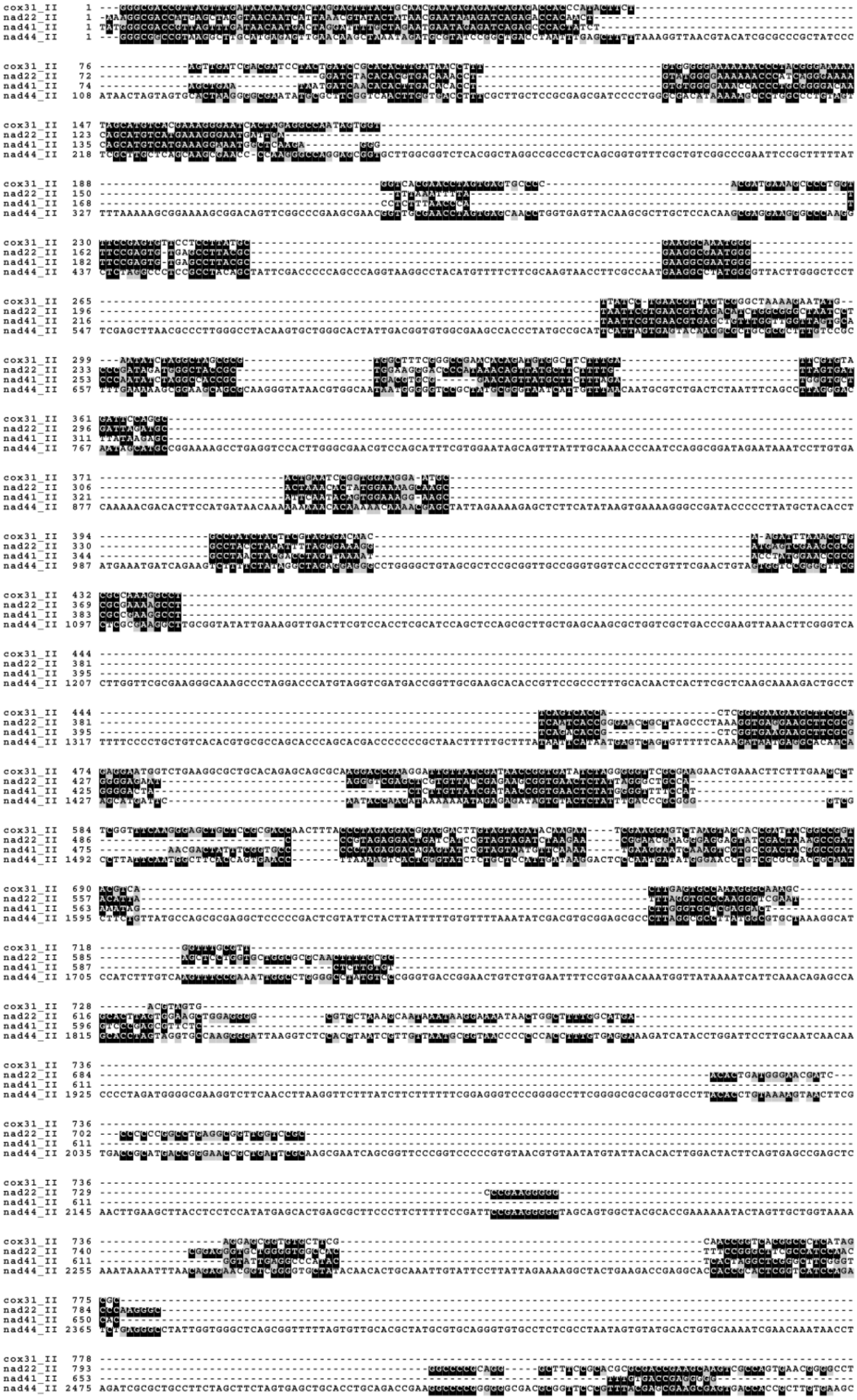

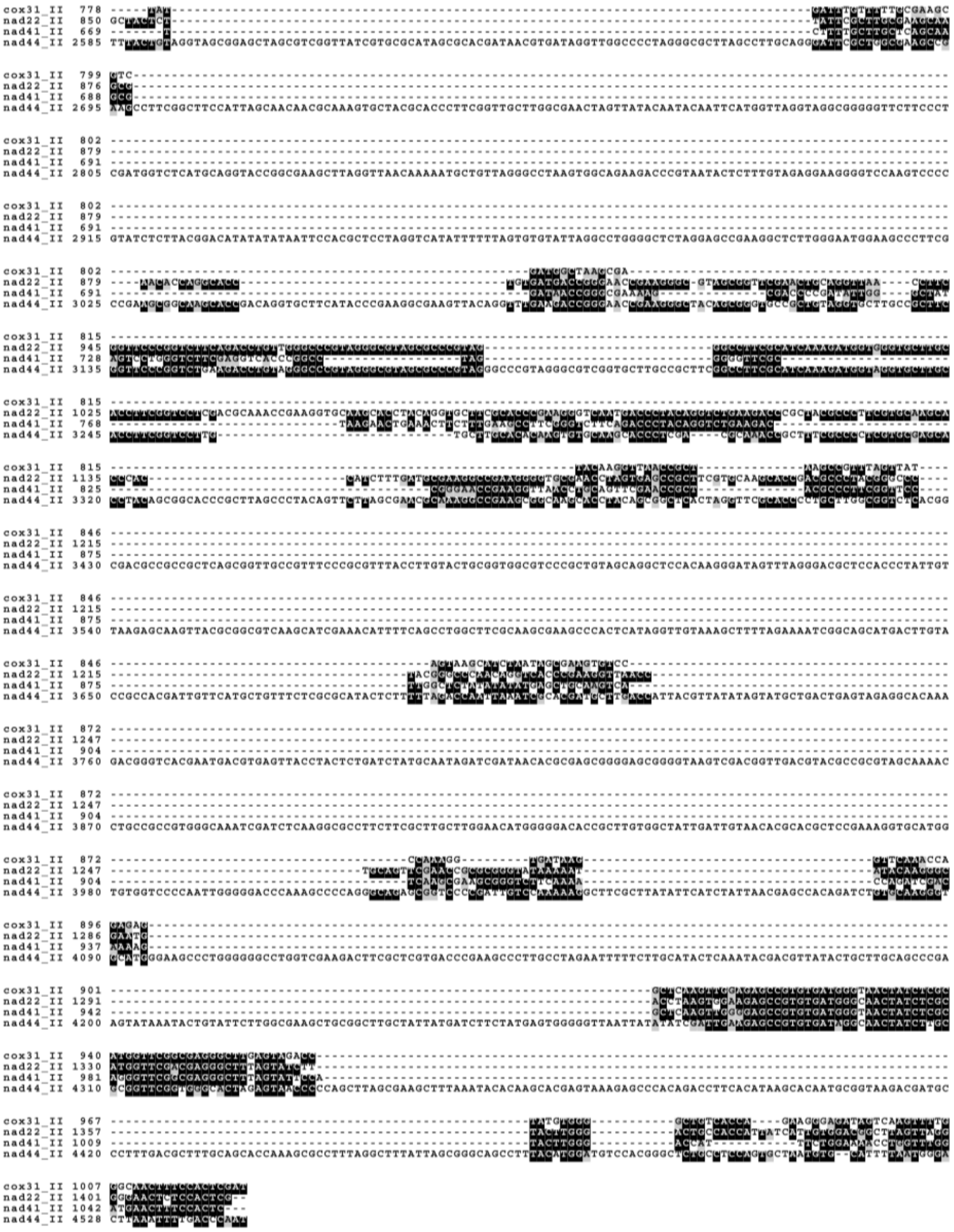

Group 3:

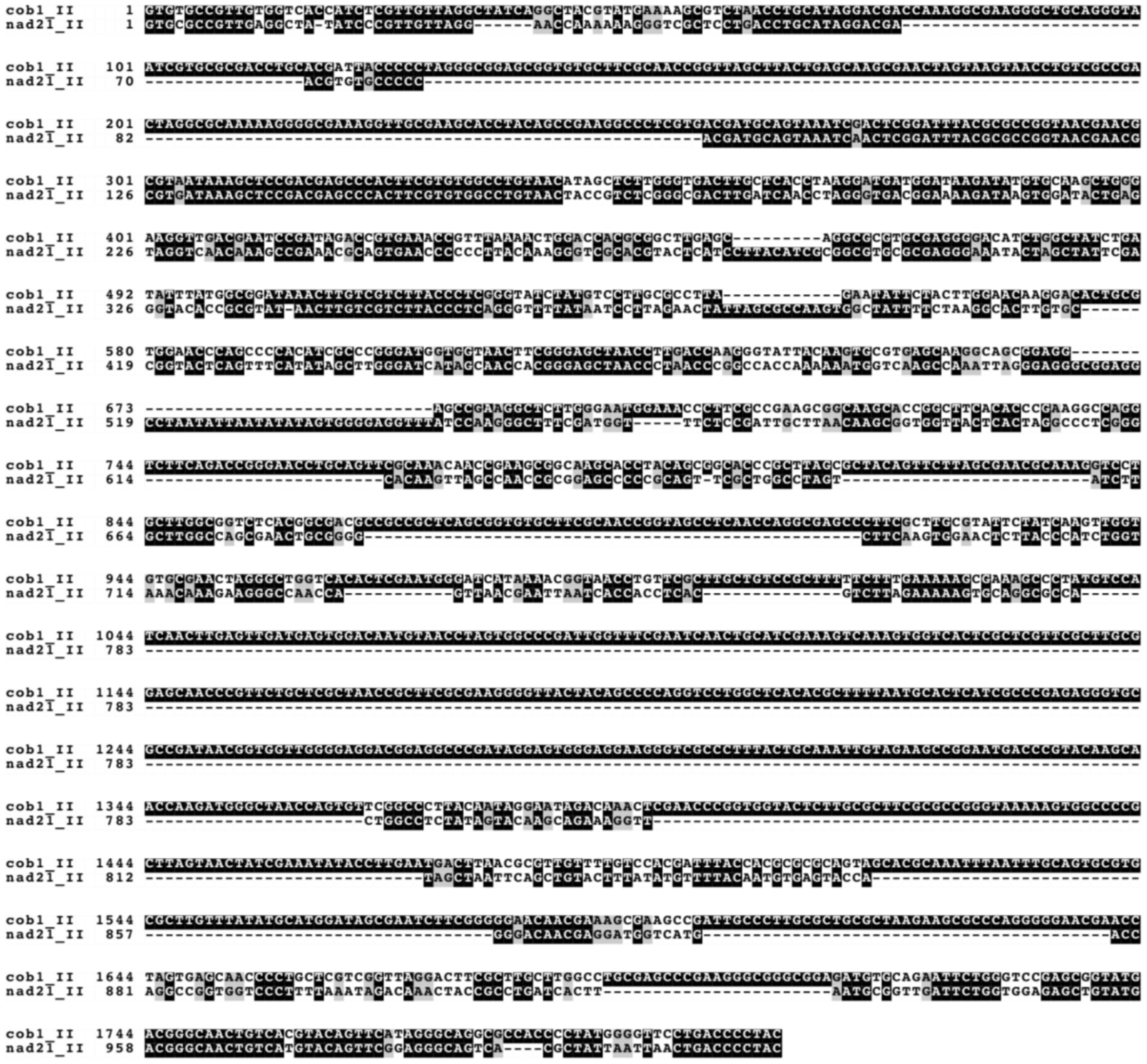

Group 4:

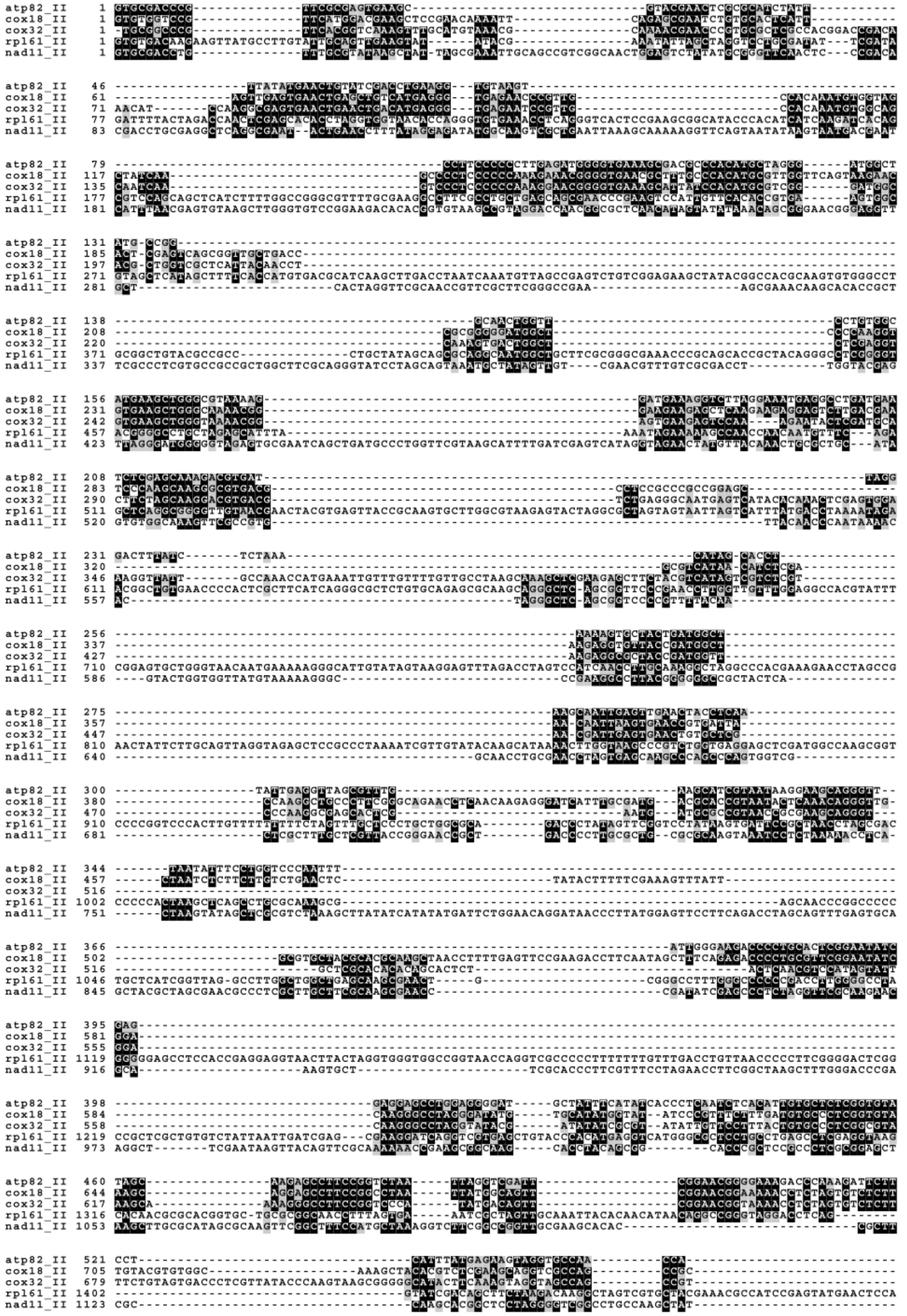

```
atp82_II   550 ATATCTCTGGGTAGTAGGATGAGATAAAAAGCTAATGCTCCCTTGTAATGAGGTA------------GATATGCTGACGTGTCTCCGCATCACATATAA-
cox18_II   751 ATGGCACTTGAGTGTAGGATGAGGTAAGAAGCTAATGCCAATCTGTAATAGATTG------------GATACGCCCACGTACCTCCGC------------
cox32_II   744 ATGGCACTTGAGAGTAGGATGAGGTAAGAAGCTAATGCCAATCTGTAATAGATTG------------GATACGCCCACGTACCTCCGCGTGTTGGGCAAA
rpl61_II  1465 GTACCATGCGGGTAGAGGACACCCTAAAAAGCGACTTTCCTTATAGGATCCGCTGTTCGCTAATATCCGTTTTTTTATGTTCTTGCTGTTCTTTTGCGTG
nad11_II  1162 ATACTCCATAGGGGCGGGATTTGCCAAAAAGCCAACGCCCTGGTGAAATACGAGG------------GATATGCTGACATGGTCACTCAGAATATTTGCA

atp82_II   637 ----------------------------------------------------------------------------------------------------
cox18_II   827 ----------------------------------------------------------------------------------------------------
cox32_II   832 AATTTTGTATATTTTATTAGCAGACGCACTCGCCACGCTCTGATCTAGACAATAATCAAGAGGCACTGCTACGCCATACTGAAT----AAT---------
rpl61_II  1565 GGTCCCTTTTGTGACACTATGCTCGGAAGCACCTAGGGCGTTTG-TATACACTAGCTATCGGGAACCGCTCCGCCCTTCCAGTTACAAAATTAACCCAAG
nad11_II  1250 GAACTAGCATGGGTCACGAACCTAGTGAGTGACCACCGCATTTGTTATTTTGCCATAACACAAGGCCCTTCTTCGCTTTCGGTTGCGAAGT---------

atp82_II   637 --------------------------TGTGCATGAATGATACTGCGCATAG---------------AGGCCTAAAAGACCTTTGAGTCTAGG--------
cox18_II   827 ----------------------------------------------------------------------------------------------------
cox32_II   919 ------GGAAAGATGGGCATTATGAGTGTGTTTGCAAGGGTGTTAGTACGG---------------TTGCGAGCAAGGTCGGGGGGTGCTGA--------
rpl61_II  1664 TTTACATAAAGTGCGCGCAGTGAGAATCAAGCACCGAGGGTCCTTGGGTGGCCACCTTCGCTTGCTTTGCAAGCGGAGACCAAGGGCGTTTGCTTAGGTC
nad11_II  1341 ------GCACGCTGGTGCGCTGGGAAAGCATCTATGTAAATTCTACACTAGCTACAAGAACAC---ATTCTGGAGAAGGCAATAGATTTTGG--------

atp82_II   688 ---------------------------------------------------------------------------------------------ATGAGGG
cox18_II   827 ----------------------------------------------------------------------------------------------------
cox32_II   990 ---------------------------------------------------------------------------------------------GCAAGCG
rpl61_II  1764 GCGCGTCATCGTGCGCGGGTAGAGCTAATTTAAATATGTTGGAATGGGTTAATTGGTTGGAGTCTCTAGATGTTTATGCAGAAAAAGTTTCATATAAGCG
nad11_II  1424 ---------------------------------------------------------------------------------------------ATAAGAA

atp82_II   695 AAATTATT----------------CATAAGTCTCG---------------------------ACATTCAGTTGTGAGCAGAATACCCAAAGCC-------
cox18_II   827 ------------AGGC---ACATGCAAAGGTGC-------------------------------------------------------------------
cox32_II   997 AAAACACTTCGCAAGC---GAACGCAAAGGCCCCGCTTGGCGG---------TCTCACGGCTAGGGCCGCCCTTCAGCGGTGTTGCTATAGCA-------
rpl61_II  1864 GAAAGCTCTTAAAACCTCTAAACAATAAGGCCCCAGTGCGCGGGTGGACATTACTCGCGCCTAGGGCCGC-------GAGTCTTGCTGCAGCAACACCCG
nad11_II  1431 GCATCGTT--------------------------------------------------------------------------------------------

atp82_II   745 ------------------------------------------GAGTTGAAGCCTCTG-------------------------------------------
cox18_II   845 ----------------------------------------------------------------------------------------------------
cox32_II  1078 ------------------------------------------GAGAGGTCCCCTTTGGGGGAGCCTTCGCCTT---------------------------
rpl61_II  1957 AGAGCGAGGCGGTTCCCGGTTAATAACACCCGGGGTTTAACTGAGAAGTCCCCGAATGGAGGGCCGTGACCTCTGATGTCTGATGTCTTATGTAGTGCAT
nad11_II  1439 ----------------------------------------------------------------------------------------------------

atp82_II   760 ----------------------------------------------------------------------------------------------------
cox18_II   845 ----------------------------------------------------------------------------------------------------
cox32_II  1109 -----------------------GCACTCTCGGGGTAACGAGCCCTTGGGTGTGCTC-------------------------------------------
rpl61_II  2057 AAGGCGCGCAATCCAGGGTGAGGTCACTGGTAACGTAGTAACCACTTATGCATGCTCCGCCTAAGTGAAACAGAGAGCAGTAAAATTTTAATGCGCTCGG
nad11_II  1439 ----------------------------------------------------------------------------------------------------

atp82_II   760 ----------------------------------------------------------------------------------------------------
cox18_II   845 ----------------------------------------------------------------------------------------------------
cox32_II  1143 -------------------------------------------------------------------------------G--------------------
rpl61_II  2157 TTGCAGACAATAGAATGATCTGCCGAGTATATAATATAGCTAAATAGTTTACATTTAGTCAGAGTTAGCACAAAAATGGTATTTGGACCATTGGGTATCT
nad11_II  1439 -----------------------------------------GGCTAATT---ACCGAGTTGGGGTGAACACCTCAATGGG--------------------

atp82_II   760 ----------------------------------------------------------------------------------------------------
cox18_II   845 ----------------------------------------------------------------------------------------------------
cox32_II  1144 -----------------------------CTTCGCTCGCTCCTGGTTATTCTGT-----------------------------------------GGCTC
rpl61_II  2257 ATATCGAATGAGCTCACTTGGGGTTGCACTTCAGGGTGCTCCTGGTAATCTTATTTTGAAGGCTCACCAATTAAGCGAGCCAGAATTAGCCAACTGGGCT
nad11_II  1475 -------------------------------------------------------------GTTCGCTTGTTAAACAAGCGAACCCCCTTCGAAAGATCT

atp82_II   760 ----------------------------------------------------------------------------------------------------
cox18_II   845 ----------------------------------------------------------------------------------------------------
cox32_II  1174 TCGTACTTTCT-----------------------------------------------------------------------------------------
rpl61_II  2357 TCGCAGGTTGTTACGCGTACATTCACTTGTTCGCTTGCTGTCCGCTTTTTAAAATAAAAAGCGGAATTCGGGGCGAAAGAAGCGCTTTTATTCGCAAGAA
nad11_II  1514 CCATAGATTGT-----------------------------TCAGATGCATAATAGAAAAAGCATGTCTTGGTGCACAATAGGGGAAGCGGTCC-CCAGTG

atp82_II   760 -----------------------------------------------------------------------------------AACCTCTTATA-CGTAA
cox18_II   845 ---------------------------------------------------------------------------------------------------C
cox32_II  1185 ---------------------------------------------------------------------------------CAAACCCTTTAC------A
rpl61_II  2457 AGCAAACACCCGAAGGCTTCGAAGAAGTTACGGGTCGAAGACCCTTCGGTTCCCGCGTCAGCTATGTAGCGCACGGCGCGACCTACATCTTCCA-TAAGA
nad11_II  1584 TGCAGGCACCCATTTACTATAAA--------------------------------------------------------AATATGCATTTACCGAAATTC

atp82_II   776 TGCGAGGAGCTGGATGAAGGGAAACTTTCACGTCCAGTTCTGAAGTAGAGGTAGTGTGGGAGACCCGCTATCGACTATAAC-
cox18_II   846 TGCGAGGAGCCGTATGACGGGAAACTGTCACGTACGGTTCTGAATTGGCGGTTAAGAGGGAGACCCTTCGCCGACCATAAC-
cox32_II  1198 CGCGAGGAGCCGTATGAGGCGAAAGTTTCACGTACGGTTTTGTATTAGAGGTGAGGTGGGAGACCCCTTACCTACTATGACG
rpl61_II  2556 TTGCTTGAGCCGTGTGTGATTAAAATCACACGCACGGTTCT---------------TCGGGGGGTTTAAACCTATCCCAAC-
nad11_II  1628 TGAGAGGAGCCGTATGAGGTAAAAGCCTCACGTACGGTTTTGAAGCCGAGCCATTTCAGCAATGCCATGGCTTAGGTTAAC-
```

Group 5:

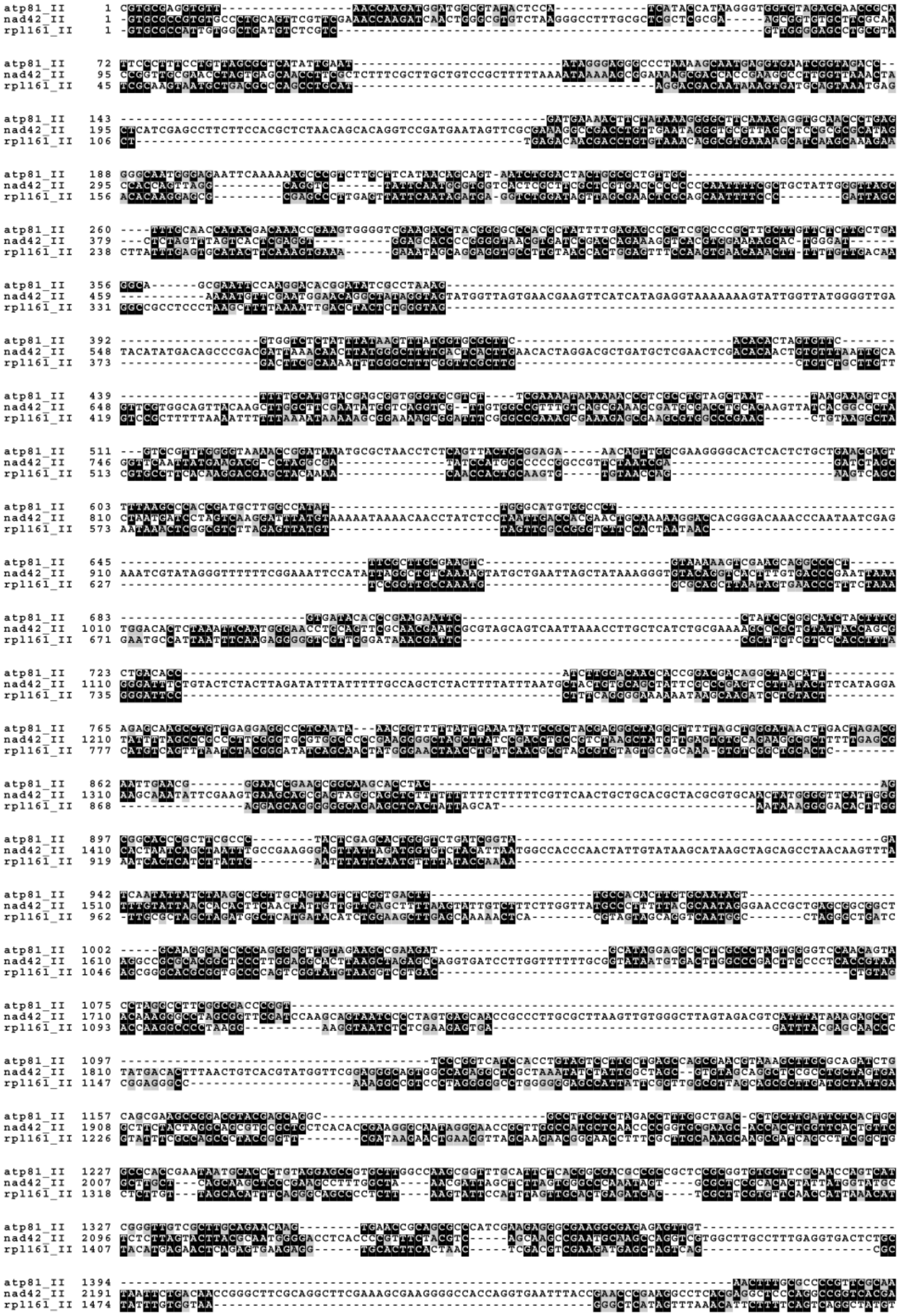

```
atp81_II  1415 AGGTAAAACCACTTCGCAAGCGAAGAATAGCTGCAGCTTGAAGTTCGCTGCATAGGTTCTGGCCTTC---------------------------------
nad42_II  2291 ATGTAAGCACCTTTGTCGAAGCTTGCTCAGCTAGCGAAGGGGCCACCGGGCTTGGCTCCTCCCCCCCCCGAAGGGTGCATGGAAAGCCGAAGGCCAAAGG
rpl161_II 1525 G-------------------------ACTTCTGACCAAGTAACCCATCATTTTGGGTCT---------------------------------GCTATAAC

atp81_II  1482 -------GACCTTCTCCAAAGGCCCCGCAG--------------------------------------------CGCTTATGTTTACTTGGTCAACATAA
nad42_II  2391 CCTACAGGTCTTCCTAAAAAGGGTTTCCAGTGGTGGCCAAGGAGCCGTGCTTGGCGGTCTCACGGCTAGGCCGCCGCTCAGCGGTTCCCGTTCAGAGCGA
rpl161_II 1567 TATACAGG------CAAAGGTGATTTTTTTTAATCACCCATTGAGTGC----------------------------CTCGGGTGTTCGCTTTCAGCCTTG

atp81_II  1531 GGCTAACGCATGAGCCTTTGGTCACGCACACTCATTATTCTACACAACGTAGGGCAACTTACTGCTAAGATGAAAATGCGTGCAAGCCGTAT--------
nad42_II  2491 GGCACTCGCTATGACATGCGAAGAAGGCCGTCCACTGATGTGTATAACCTCGATAGAC------CGCGAAGGGCATTCCATGTAAGCCCGATCGCCCGGC
rpl161_II 1633 GCCAC-----------TATAACATAGAATGTTCGGTGGCGTGACCGCCACAAAGAGAT------CGCAGGCGGAG---------AGCCGTAT--------

atp81_II  1623 ------------------------------------------GATGGGTAACTATCACGTACGGTTACGAGAAGGGGGCCCTGAGGGCGCCGCAATGGTG
nad42_II  2585 ACCTTCACTCAACCGAAGTCCACAAGACCACCCCGAAGGGCGAAGCGGTGTGCTTCGCAACCGGTTGCGAACCTGGTG-------AGCAACCTATCTGCG
rpl161_II 1699 ------------------------------------------GAACGATAAATTTCATGTACGGTTCGGAGGGCAGTA----------------------

atp81_II  1681 ATTACCCGTT-----GTGATTAGCCGGAAGGGTTTCTATTCTA-----------------------
nad42_II  2678 GTTATCCACTACTAGGTGGCTAACCAGCCGTATAAACGGTGCCTCGGCCCCTGACCCCTAC
rpl161_II 1735 -----------------------------------------------CCACTGACCCCCAT
```
