# Supplementary material for: The inflated mitochondrial genomes of siphonous green algae reflect processes driving expansion of noncoding DNA and proliferation of introns
Source: PeerJ. 2020 Jan 3;8:e8273. doi: 10.7717/peerj.8273 (PMC6944098; doi:10.7717/peerj.8273)
Supplement: Supplemental Information 4 [file peerj-08-8273-s004.docx]

| **Table S1: Codon usage in the protein coding genes in mtDNA of *Ostreobium quekettii* SAG6.99.** | | | | | | | | | | | |
| --- | --- | --- | --- | --- | --- | --- | --- | --- | --- | --- | --- |
| **Codon** | **AA** | **% of AA** | **Freq** | **Codon** | **AA** | **% of AA** | **Freq** | **Codon** | **AA** | **% of AA** | **Freq** |
| GCA | A | 26.30% | 390 | AAC | N | 35.60% | 363 | TTG | M | 0.60% | 3 |
| GCC | A | 23.50% | 348 | AAT | N | 64.40% | 656 | TGG | W | 100.00% | 323 |
| GCG | A | 17.90% | 266 | CCA | P | 27.00% | 263 | TAG | * | 28.80% | 19 |
| GCT | A | 32.30% | 478 | CCC | P | 21.70% | 211 | TGA | * | 19.70% | 13 |
| TGC | C | 43.10% | 178 | CCG | P | 14.10% | 137 |  | | | |
| TGT | C | 56.90% | 235 | CCT | P | 37.20% | 362 |  |  |  |  |
| GAC | D | 34.10% | 331 | CAA | Q | 71.90% | 634 |  |  |  |  |
| GAT | D | 65.90% | 641 | CAG | Q | 28.10% | 248 |  |  |  |  |
| GAA | E | 72.20% | 721 | AGA | R | 22.70% | 384 |  |  |  |  |
| GAG | E | 27.80% | 278 | AGG | R | 11.80% | 200 |  |  |  |  |
| TTC | F | 32.50% | 440 | CGA | R | 20.40% | 345 |  |  |  |  |
| TTT | F | 67.50% | 915 | CGC | R | 15.00% |  |  |  |  |  |
| GGA | G | 27.20% | 360 | CGG | R | 11.20% |  |  |  |  |  |
| GGC | G | 20.60% | 273 | CGT | R | 18.80% | 317 |  |  |  |  |
| GGG | G | 18.30% | 242 | AGC | S | 15.20% | 280 |  |  |  |  |
| GGT | G | 33.90% | 449 | AGT | S | 19.10% | 352 |  |  |  |  |
| CAC | H | 37.10% | 239 | TCA | S | 18.60% | 344 |  |  |  |  |
| CAT | H | 62.90% | 406 | TCC | S | 10.50% | 194 |  |  |  |  |
| ATA | I | 35.10% | 638 | TCG | S | 13.90% | 257 |  |  |  |  |
| ATC | I | 20.50% | 373 | TCT | S | 22.70% | 418 |  |  |  |  |
| ATT | I | 44.50% | 809 | ACA | T | 29.00% | 340 |  |  |  |  |
| AAA | K | 69.40% | 1193 | ACC | T | 24.00% | 281 |  |  |  |  |
| AAG | K | 30.60% | 525 | ACG | T | 13.10% | 154 |  |  |  |  |
| CTA | L | 14.50% | 377 | ACT | T | 33.90% | 397 |  |  |  |  |
| CTC | L | 9.60% | 249 | GTA | V | 27.80% | 383 |  |  |  |  |
| CTG | L | 8.90% | 232 | GTC | V | 17.70% | 244 |  |  |  |  |
| CTT | L | 19.80% | 515 | GTG | V | 23.70% | 327 |  |  |  |  |
| TTA | L | 28.90% | 750 | GTT | V | 30.70% | 423 |  |  |  |  |
| TTG | L | 18.20% | 473 | TAC | Y | 35.10% | 348 |  |  |  |  |
| ATG | M | 99.00% | 482 | TAT | Y | 64.90% | 643 |  |  |  |  |
| CTG | M | 0.40% | 2 | TAA | * | 51.50% | 34 |  |  |  |  |
