# Supplementary material for: The inflated mitochondrial genomes of siphonous green algae reflect processes driving expansion of noncoding DNA and proliferation of introns
Source: PeerJ. 2020 Jan 3;8:e8273. doi: 10.7717/peerj.8273 (PMC6944098; doi:10.7717/peerj.8273)
Supplement: Supplemental Information 5 [file peerj-08-8273-s005.docx]

| **Table S2: tRNAs present in mtDNA of Ostreobium quekettii SAG6.99.** | | | | |
| --- | --- | --- | --- | --- |
| **trna** | **start** | **stop** | **#nt** | **direction** |
| tRNA-Lys(uuu) | 26,865 | 26,937 | 73 | forward |
| tRNA-Glu(uuc) | 27,231 | 27,302 | 72 | forward |
| tRNA-Met(cau) | 73,803 | 73,875 | 73 | forward |
| tRNA-Met(cau) | 84,822 | 84,893 | 72 | forward |
| tRNA-Ala(ugc) | 86,936 | 87,008 | 73 | forward |
| tRNA-Ile(gau) | 92,048 | 92,121 | 74 | forward |
| tRNA-Ser(uga) | 92,893 | 92,978 | 86 | forward |
| tRNA-Ser(gcu) | 95,358 | 95,445 | 88 | forward |
| tRNA-Gly(ucc) | 98,148 | 98,218 | 71 | forward |
| tRNA-Leu(caa) | 99,103 | 99,188 | 86 | forward |
| tRNA-Thr(gag) | 113,591 | 113,672 | 82 | forward |
| tRNA-Thr(uag) | 139,600 | 139,679 | 80 | forward |
| tRNA-Pro(ugg) | 142,712 | 142,787 | 76 | reverse |
| tRNA-His(gug) | 159,029 | 159,100 | 72 | reverse |
| tRNA-Arg(ucu) | 179,218 | 179,291 | 74 | forward |
| tRNA-Asn(guu) | 180,642 | 180,713 | 72 | forward |
| tRNA-Trp(cca) | 187,710 | 187,781 | 72 | forward |
| tRNA-Asp(guc) | 191,017 | 191,089 | 73 | forward |
| tRNA-Arg(acg) | 191,095 | 191,168 | 74 | forward |
| tRNA-Gly(gcc) | 193,289 | 193,360 | 72 | forward |
| tRNA-Gln(uug) | 204,895 | 204,965 | 71 | forward |
| tRNA-Met(cau) | 217,806 | 217,877 | 72 | forward |
| tRNA-Cys(gca) | 221,376 | 221,447 | 72 | forward |
| tRNA-Thr(ugu) | 223,196 | 223,268 | 73 | forward |
| tRNA-Tyr(gua) | 223,271 | 223,352 | 82 | forward |
| tRNA-Leu(uaa) | 226,169 | 226,249 | 81 | forward |
| tRNA-Val(uac) | 234,890 | 234,962 | 73 | forward |
| tRNA-Phe(gaa) | 237,211 | 237,284 | 74 | forward |
