# Supplementary material for: The inflated mitochondrial genomes of siphonous green algae reflect processes driving expansion of noncoding DNA and proliferation of introns
Source: PeerJ. 2020 Jan 3;8:e8273. doi: 10.7717/peerj.8273 (PMC6944098; doi:10.7717/peerj.8273)
Supplement: Supplemental Information 8 [file peerj-08-8273-s008.docx]

| **Table S5**: **Estimates of base substitutions per site between genes from *Ostreobium quekettii* and *Caulerpa lentillifera* using the Jukes Cantor model conducted in MEGA.** | | | | | | | | | |
| --- | --- | --- | --- | --- | --- | --- | --- | --- | --- |
| **Mitochondrion** | | **Chloroplast** | | | | | | | |
| *atp*6 | 0.562 | *atp*I | 0.290 | *pet*B | 0.238 | *psb*L | 0.171 | *rps*4 | 0.538 |
| *atp*9 | 0.296 | *atp*H | 0.191 | *pet*D | 0.214 | *psb*M | 0.254 | *rps*7 | 0.463 |
| *atp*1 | 0.425 | *atp*A | 0.297 | *pet*G | 0.268 | *psb*N | 0.243 | *rps*8 | 0.460 |
| *atp*8 | 0.556 | *atp*F | 0.383 | *pet*L | 0.467 | *psb*T | 0.202 | *rps*9 | 0.743 |
| *cob* | 0.396 | *atp*E | 0.382 | *psa*A | 0.246 | *psb*Z | 0.352 | *rps*11 | 0.555 |
| *cox*1 | 0.348 | *atp*B | 0.273 | *psa*B | 0.246 | *rbc*L | 0.219 | *rps*12 | 0.326 |
| *cox*2 | 0.531 | *rrn*5 | 0.531 | *psa*C | 0.204 | *rpl*2 | 0.464 | *rps*14 | 0.417 |
| *cox*3 | 0.467 | *rrn*S | 0.198 | *psa*I | 0.321 | *rpl*5 | 0.502 | *rps*18 | 0.629 |
| *rrn*5 | 0.441 | *rrn*L | 0.267 | *psa*J | 0.310 | *rpl*14 | 0.349 | *rps*19 | 0.434 |
| *rrn*S | 0.285 | *acc*D | 0.537 | *psa*M | 0.339 | *rpl*16 | 0.433 | *tuf*A | 0.276 |
| *rrn*L | 0.490 | *chl*B | 0.248 | *psb*A | 0.245 | *rpl*19 | 0.418 | *ycf*1 | 0.633 |
| *nad*1 | 0.342 | *chl*I | 0.407 | *psb*B | 0.278 | *rpl*20 | 0.479 | *ycf*3 | 0.278 |
| *nad*2 | 0.485 | *chl*L | 0.246 | *psb*C | 0.243 | *rpl*23 | 0.402 | *ycf*4 | 0.520 |
| *nad*3 | 0.499 | *chl*N | 0.338 | *psb*D | 0.211 | *rpl*32 | 0.503 | *ycf*20 | 0.588 |
| *nad*4 | 0.679 | *clp*P | 0.318 | *psb*E | 0.248 | *rpl*36 | 0.244 |  | |
| *nad*4L | 0.364 | *cys*A | 0.397 | *psb*F | 0.241 | *rpo*A | 0.687 |  |  |
| *nad*5 | 0.635 | *cys*T | 0.525 | *psb*H | 0.342 | *rpo*C1 | 0.707 |  |  |
| *nad*6 | 0.365 | *fts*H | 0.477 | *psb*I | 0.246 | *rpo*C2 | 0.426 |  |  |
| *nad*7 | 0.384 | *inf*A | 0.38 | *psb*J | 0.267 | *rps*2 | 0.448 |  |  |
| *nad*9 | 0.568 | *pet*A | 0.364 | *psb*K | 0.388 | *rps*3 | 0.393 |  |  |
